# Cortical involvement in essential tremor with and without rest tremor: a machine learning study

**DOI:** 10.1007/s00415-023-11747-6

**Published:** 2023-05-05

**Authors:** Maria Giovanna Bianco, Andrea Quattrone, Alessia Sarica, Federica Aracri, Camilla Calomino, Maria Eugenia Caligiuri, Fabiana Novellino, Rita Nisticò, Jolanda Buonocore, Marianna Crasà, Maria Grazia Vaccaro, Aldo Quattrone

**Affiliations:** 1 Department of Medical and Surgical Sciences, Neuroscience Research Center, University “Magna Graecia”, Catanzaro, Italy; 2 Institute of Neurology, Department of Medical and Surgical Sciences, Magna Graecia University, Catanzaro, Italy

**Keywords:** Essential tremor plus, Rest tremor, Machine learning, Cortical thickness, Roughness

## Abstract

**Introduction:**

There is some debate on the relationship between essential tremor with rest tremor (rET) and the classic ET syndrome, and only few MRI studies compared ET and rET patients. This study aimed to explore structural cortical differences between ET and rET, to improve the knowledge of these tremor syndromes.

**Methods:**

Thirty-three ET patients, 30 rET patients and 45 control subjects (HC) were enrolled. Several MR morphometric variables (thickness, surface area, volume, roughness, mean curvature) of brain cortical regions were extracted using Freesurfer on T1-weighted images and compared among groups. The performance of a machine learning approach (XGBoost) using the extracted morphometric features was tested in discriminating between ET and rET patients.

**Results:**

rET patients showed increased roughness and mean curvature in some fronto-temporal areas compared with HC and ET, and these metrics significantly correlated with cognitive scores. Cortical volume in the left pars opercularis was also lower in rET than in ET patients. No differences were found between ET and HC. XGBoost discriminated between rET and ET with mean AUC of 0.86 ± 0.11 in cross-validation analysis, using a model based on cortical volume. Cortical volume in the left pars opercularis was the most informative feature for classification between the two ET groups.

**Conclusion:**

Our study demonstrated higher cortical involvement in fronto-temporal areas in rET than in ET patients, which may be linked to the cognitive status. A machine learning approach based on MR volumetric data demonstrated that these two ET subtypes can be distinguished using structural cortical features.

**Supplementary Information:**

The online version contains supplementary material available at 10.1007/s00415-023-11747-6.

## Introduction

Essential tremor (ET) is one of the most common neurological disorder, with a high prevalence in the general population [1]. The core symptom of ET is symmetric action tremor in the upper limbs, with possible presence of tremor in the head, tongue, torso, jaw, legs or voice [2]. A recent consensus statement coined the construct “ET plus” for ET patients presenting with additional motor and non-motor features, such as impaired tandem gait, cognitive impairment or questionable dystonic posturing/parkinsonian features [2]. According to the recent tremor consensus, ET patients with rest tremor (rET) should be included in the ET plus group [2]. The new classification has the main advantage of defining the entity of a “pure” ET syndrome, moving patients with additional symptoms to the “ET plus” category. This change, however, has also found criticism and controversy, since it is not yet clear whether ET plus represents an advanced disease stage of ET or a different condition [3–6].

Recent clinical studies provided evidence that ET plus may be even more common than the classic ET, and rest tremor is one of the most common symptoms in ET plus cohorts [7–9]. However, only few MRI studies compared ET and rET patient groups so far. Most of these MRI studies focused on the cerebellum [10–12], and data are consistent across different reports showing no differences between ET and rET in this region [11–13]; on the contrary, some differences have been reported in the basal ganglia circuits, especially involving the globus pallidus internus [13, 14]. A couple of functional MRI (fMRI) studies [13, 15] suggested decreased activation of cortical regions in rET compared with ET patients, but no MRI study deeply investigated structural cortical differences between these two ET subtypes.

Moreover, no study explored the possible role of MR structural data in supporting the differential diagnosis between ET patients with and without rest tremor. The classification is clinically guided by the presence/absence of rest tremor, but this sign may fluctuate over time and be not always detectable during clinical assessment, making the differential diagnosis at times challenging [9]. Recently, machine learning approaches in medicine have gained a huge interest as helpful tool in the differential diagnosis and to guide clinical decision making [16, 17]. Moreover, ML algorithms take in account non-linear and high dimensional relationships among variables and are able to identify the measures that help most in the classification of patients. Several machine learning algorithms, including linear models, kernel-based model (SVM), ensemble learning model (i.e., random forest and XGBoost), and neural network models have been recently applied on structural MRI features in the differential diagnosis of various neurological diseases [17–21].

In this study, we aimed to explore differences between ET with and without rest tremor in multiple MRI-derived cortical morphometric measures (thickness, volume, surface area, mean curvature and roughness) and subcortical volumes, to improve the knowledge of these tremor syndromes. In addition, we investigated whether XGBoost, which is a powerful machine learning decision-tree-based ensemble algorithm using eXtreme Gradient Boosting to maximize the classification performance, could help discriminate between these two ET subtypes using on structural MRI data.

## Methods

### Participants

Sixty-three ET patients (30 with and 33 without rest tremor) and 45 control subjects were consecutively recruited at the Institute of Neurology at the University Magna Graecia of Catanzaro, Italy between 2017 and 2021. All patients underwent a detailed neurological examination performed by a movement disorder specialist, and the clinical diagnosis of ET or rET (now included in the “ET plus” category) was performed according to the recent consensus statement of the Movement Disorder Society task force [2]. In addition, all patients underwent surface electromyographic tremor analysis as previously described [22, 23] to confirm or exclude the presence of rest tremor, and all rET and ET patients had normal tracer uptake on single photon emission computed tomography with 123I-ioflupane (DaTscan), performed as previously described [24]. A battery of neuropsychiatric tests was administered by an experienced neuropsychologist, including: Mini Mental State Examinations (MMSE) for general cognitive impairment; the Rey Auditory Verbal Learning Test immediate (RAVLT_I) and delayed recalls (RAVLT_D), used to assess verbal learning and memory; the Controlled Oral Word Association Test (COWAT), used as a measure of lexical stock; the Digit Span Forwards (Digit Span_F) and Backwards (Digit Span_B) used to assess the short-term verbal memory. No patients had dysmetabolic causes of tremor such as thyroid dysfunction, other degenerative neurological diseases, or intracranial lesions. No patients were on medications with potential tremor-enhancing properties (e.g., amiodarone, amphetamines, beta-adrenergic agonists, antipsychotics, prednisone, lithium, and valproate). None of the control subjects had a history of neurological, psychiatric, or other major medical illnesses. According to the Helsinki Declaration, all participants gave written informed consent, which was approved by the local institutional ethical committee.

### MRI acquisition

All MRI scans were performed with the same 3-T MR750 General Electric scanner with a 8-channel head coil (Discovery MR- 750, GE, Milwaukee, WI, USA) and a recently described protocol [25].

### Image processing and feature extraction

The automated neuroanatomical segmentation was performed with FreeSurfer 6.0 software, (Massachusetts General Hospital, Harvard Medical School; http://surfer.nmr.mgh.harvard.edu) in all study participants. The following morphometric metrics were calculated using surface-based and volume-based methodologies into 34 cortical regions of interest (ROIs) per hemisphere according to the Desikan–Killiany atlas: cortical thickness (CT), surface area (SA), cortical volume (CV), mean curvature (MC) and roughness (RG; the standard deviation of cortical thickness) [21, 26]. Subcortical structures (cerebellum, thalamus, caudate, putamen, globus pallidus, hippocampus, amygdala and nucleus accumbens) were also segmented to obtain volumetric data. A total of 358 structural features were extracted from each subject. The reconstruction and surface extraction results obtained using the freesurfer pipeline were validated by visual inspection performed independently by two trained raters to exclude the presence of artifact and inaccurate segmentation.

### Statistical analysis

Statistical analyses were performed using R statistical sofware (R for Unix/Linux, version 4.1.2, the R Foundation for Statistical Computing, 2014). Normality of data distribution was checked with Shapiro–Wilk test. Fisher’s exact test was employed to assess differences in sex distribution. Age at examination and education level were compared among subjects using an analysis of variance (ANOVA), followed by post-hoc test. Mann Whitney Wilcoxon Test was used to test differences of age at disease onset and disease duration between the two groups of patients. An analysis of covariance (ANCOVA) was applied to compare cognitive scores and imaging data among groups with age, sex and education level as covariates of no interest. Further analysis of covariance was applied on structural MRI using MMSE as covariates since it was significant among groups. Partial linear correlations between cognitive performance and structural imaging metrics with age and education level as covariates were evaluated with Spearman's test. All statistical analyses were corrected for multiple comparisons (Bonferroni’s correction) and a p-level < 0.05 was considered as significant.

### Classification using XGBOOST model

The multivariate XGBoost classifier (https://xgboost.readthedocs.io/en/stable/) was used to discriminate among groups using the structural features extracted from T1-weighted MR images [27]. The framework was implemented in Python 3.9 and scikit-learn 1.0.1. For each comparison (rET versus ET, rET versus controls, ET versus controls), we trained 55 models resulting from different combination of structural metrics. For each model, the hyperparameter tuning with Random Search (100 iterations) was performed on a fivefold cross-validation dataset to optimize the classifier parameters. Subsequently, permutation feature importance procedure was applied to provide information about the most informative features. Finally, repeated stratified fivefold cross-validation (repeated 5 times) was used to get an even more robust estimation of machine learning models’ performance. The classification performances of XGBoost models were evaluated with receiver operating characteristic (ROC) analysis, and the area under the curve (AUC), accuracy, sensitivity and specificity of the model were calculated.

## Results

### Demographic and clinical features

Demographic, clinical, and neuropsychological data of patients and controls are shown in Table 1. No differences were found between ET and rET patients in age, sex and disease duration. rET patients had lower education level than the other groups, thus all the analyses were corrected for this variable. rET group showed a slightly lower MMSE score than controls, without marked involvement of other neuropsychological tests.

**Table 1:** Demographic, clinical and imaging data of patients with essential tremor with and without rest tremor, and control subjects

Data	ET with rest tremor (*N* = 30)	ET without rest tremor (*N* = 33)	Control subjects (*N* = 45)	*p*-value
Sex (M/F)	15/15	17/16	27/18	0.6^a^
Age at examination (years)^b^	65.5 ± 10.2	62.7 ± 11.4^*^	68.5 ± 6.92	0.03^c^
Age at disease onset (years)^b^	48.7 ± 16.4	50.7 ± 16.4	-	0.6^d^
Education (years)^b^	8.03 ± 3.02^+^	10.5 ± 4.56	10.9 ± 4.29	0.01^c^
Disease duration (years)^b^	16.4 ± 12.9	12.4 ± 10.4	-	0.2^d^
MMSE	25.6 ± 2.57^+^	26.6 ± 2.4	27.6 ± 2.1	0.01^e^
COWAT/FAS	22.7 ± 6.75	25.5 ± 6.01	26.1 ± 10.2	0.6^e^
RAVLT R.I	36.1 ± 8.50	37.6 ± 7.17	34.8 ± 8.59	0.6^e^
RAVLT R.D	6.86 ± 2.78	7.18 ± 2.53	6.25 ± 2.41	0.5^e^
DIGIT-FW	4.95 ± 0.79	4.93 ± 0.83	5.04 ± 0.88	0.8^e^
DIGIT-BW	3.07 ± 0.70	3.15 ± 0.98	3.25 ± 0.84	0.8^e^
BECK-II	11.4 ± 6.09	10.2 ± 6.28	8.50 ± 6.06	0.6^e^

All rET patients had rest tremor; regarding other soft signs, 12 patients had subtle parkinsonian signs, 7 patients had mild memory deficits, 6 patients had impaired tandem gait and 2 patients had questionable dystonic posturing. The full cognitive battery was available in 23 rET, 22 ET and 35 control subjects

*rET* essential tremor with rest tremor, *ET* essential tremor

^a^Fishers exact test

^b^Data are expressed as mean ± standard deviation

^c^ANOVA followed by Bonferroni post-hoc test

^d^Mann Whitney Wilcoxon Test

^e^ANCOVA followed by Bonferroni post-hoc test (covariates: age, sex, education)

^*^ET vs CTRL p-value < 0.05

^+^rET vs CTRL p-value < 0.05

### Cortical and subcortical morphometric features

**Fig. 1:**
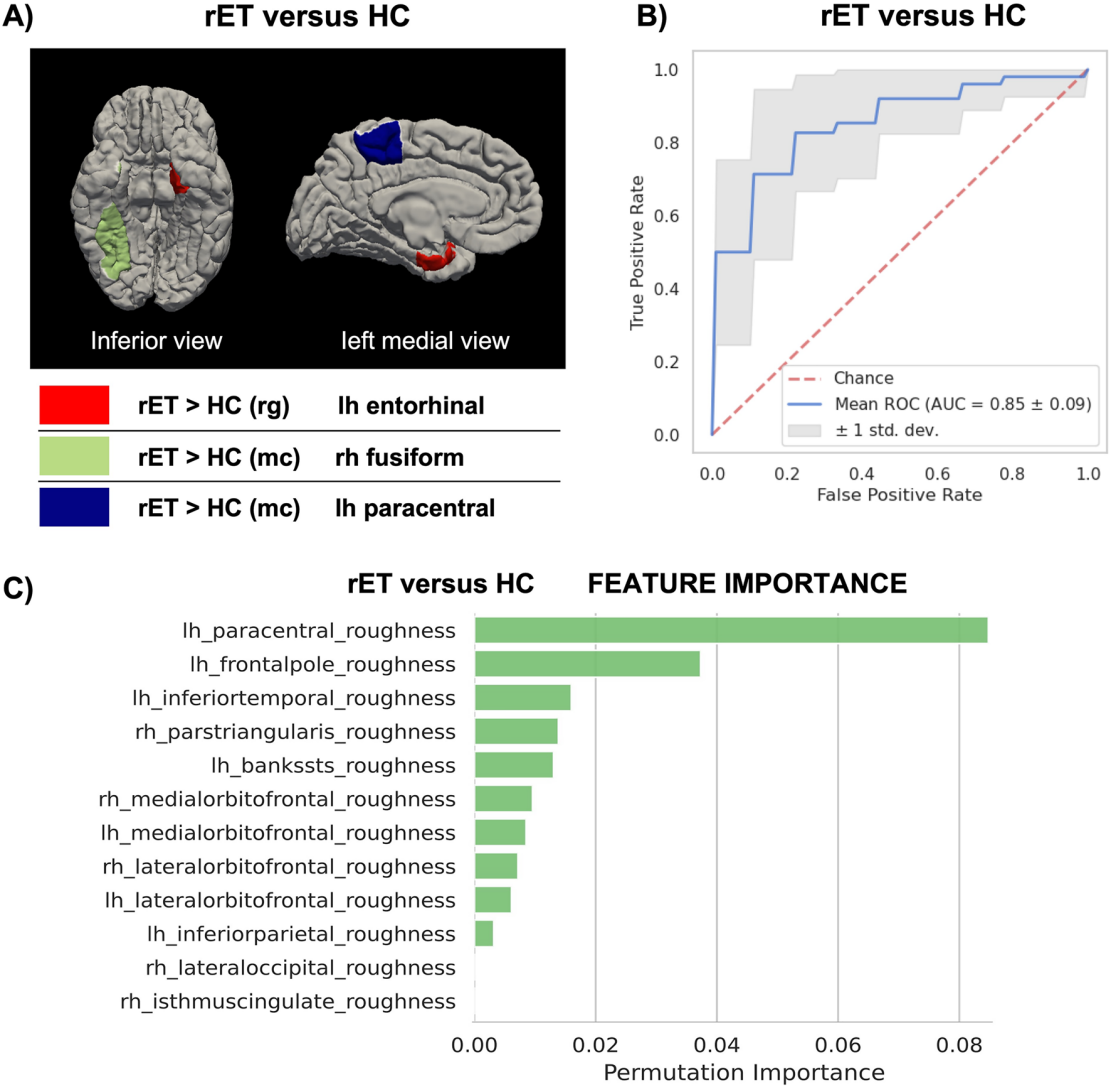
Comparison between Essential Tremor patients with rest tremor and control subjects. **A** Cortical regions showing statistically significant differences (*p* < 0.05, Bonferroni corrected) in MRI structural metrics between the two groups are highlighted in the figure. **B** Classification performance of the best XGBoost model, trained on MR metrics of cortical roughness, ranked by the permutation approach. **C** Feature importance assessed via permutation methods in distinguishing between the two groups. Data are shown in descending order from the most to the less important feature. *HC* healthy control subjects, *rET* essential tremor with rest tremor, *mc* mean curvature, *rg* roughness, *AUC* area under the curve

ET patients with rest tremor (rET) showed increased roughness and mean curvature in some temporal and frontal areas in comparison with control subjects (increased roughness in the left entorhinal cortex and increased mean curvature in the right fusiform and left paracentral cortex) (Fig. 1A and supplementary table 1). These metrics showed significant negative linear correlations with cognitive scores in the rET group. More in detail, the bilateral parahippocampal roughness, the left paracentral and the entorhinal mean curvature showed significant negative correlations, correcting for age and education level, with COWAT/FAS test in the rET group (supplementary table 2). On the contrary, no significant correlations were found between imaging data and rest tremor features (supplementary table 3).

**Fig. 2:**
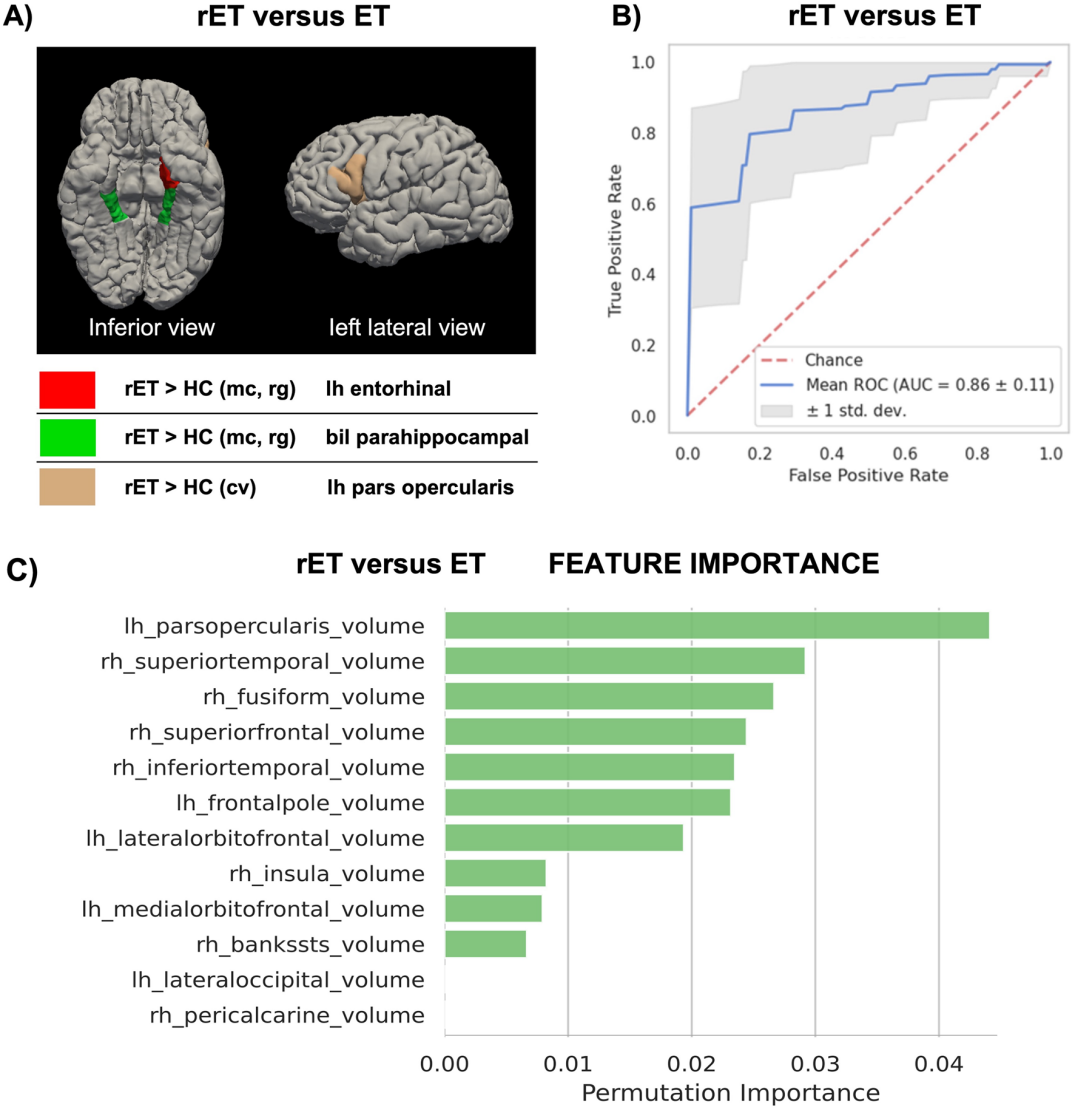
Comparison between Essential Tremor patients with and without rest tremor. **A** Cortical regions showing statistically significant differences (*p* < 0.05, Bonferroni corrected) in MRI structural metrics between the two groups are highlighted in the figure. **B** Classification performance of the best XGBoost model, trained on MR metrics of cortical volume, ranked by the permutation approach. **C** Feature importance assessed via permutation methods in distinguishing between the two groups. Data are shown in descending order from the most to the less important feature. *ET* essential tremor, *rET* essential tremor with rest tremor, *mc* mean curvature, *rg* roughness, *cv* cortical volume, *AUC* area under the curve

Differently from patients with rET, those with classic ET syndrome showed no differences in the considered metrics (cortical thickness, surface area, cortical volume, roughness and mean curvature) in any cortical region compared with control subjects. By directly comparing the two ET syndromes, rET patients showed increased mean curvature in the left entorhinal cortex, confirming the involvement of this region in rET (Fig. 2A and supplementary table 1). In addition, rET patients had higher roughness and mean curvature in parahippocampal cortex and lower cortical volume in the left pars opercularis in comparison with ET patients. Differently from cortical regions, no differences were found in subcortical structures volume among groups.

### eXtreme gradient boosting (XGBoost)

Numerous different XGBoost models (55 models for each comparison) using alternative combinations of MRI structural metrics were tested to differentiate among groups (Supplementary table 4 and 5). The model obtaining the best performance in discriminating between rET and controls was based on cortical roughness metrics, showing mean AUC of 0.850 ± 0.09 (Accuracy: 0.81 ± 0.09, Sensibility: 0.69 ± 0.20, Specificity: 0.89 ± 0.11) in cross-validation analysis (Fig. 1B). On the other hand, the best model in distinguishing between rET and ET patients was based on cortical volume metrics, showing mean AUC of 0.865 ± 0.11 (Accuracy: 0.81 ± 0.11, Sensibility: 0.78 ± 0.19, Specificity: 0.834 ± 0.13) in cross-validation analysis (Fig. 2B). Feature importance analysis identified the cortical volume in the left pars opercularis as the most informative feature for classification between the two ET syndromes (Fig. 2C). This result was in line with the statistical univariate approach which identified significantly lower cortical volume in this cortical region in rET than in ET patients. None of the models showed acceptable (> 80%) accuracy in distinguishing between ET patients and controls, in agreement with the lack of differences between these groups in the statistical univariate approach.

## Discussion

In this study, we investigated many structural MRI morphometric measures in ET patients with and without rest tremor and healthy controls, and we found higher cortical involvement (increased roughness and mean curvature) in some fronto-temporal areas in rET compared with ET and control subjects, correlating with cognitive scores. In addition, rET patients had lower cortical volume in left pars opercularis in comparison with ET patients. A machine learning model using MR morphometric metrics demonstrated that these two ET subtypes can be distinguished based on cortical structural features.

A high percentage of patients fulfilling clinical criteria for ET also show rest tremor in addition to the bilateral action tremor and are classified as “ET plus” [2]. The distinction of rET from ET, however, is considered arbitrary by some authors due to the lack of pathological or prognostic differences between these two tremor syndromes, making it possible to hypothesize that ET plus is an advanced stage of ET [3–6]. To date, the exact nature of ET with rest tremor and its relationship with classic ET are extremely controversial concepts. From the electrophysiological perspective, rET patients show enhanced R2 component of the recovery cycle of the blink reflex (R2BRrc), which is normal in ET patients without rest tremor [28]. This finding, together with the synchronous contraction pattern of rest tremor observed in rET patients [24] suggested that the rest tremor in ET plus might have some dystonic features [28], and supported the distinction of rET from “pure” ET. From the neuroimaging point of view, a few studies investigated the presence of structural and functional differences between ET patients with and without rest tremor. Most studies agreed on a similar involvement of cerebellum in ET and rET patients [10–13], and on the involvement of basal ganglia circuits in rET [13, 14] but not in classic ET syndrome, thus leading to the hypothesis that the rest tremor may be linked to these latter structures [3, 13, 14]. In the current study, we evaluated subcortical structures’ volume, and we did not find any difference between rET and ET patients in basal ganglia or cerebellar volume. This result, considered together with previous findings, suggests that a network dysfunction rather than macroscopic atrophy of basal ganglia may be involved in the pathophysiology of rest tremor in ET syndrome.

A couple of functional MRI studies found differences between ET and rET in cortical structures, with one resting-state MRI study [15] showing decreased neural activities in secondary motor cortex (right superior and middle frontal gyri, right precentral gyrus and right Supplementary motor area) and another one [13] showing decreased activation in parietal areas in rET compared to ET patients. No study, however, specifically focused on structural differences between rET and ET patients in cortical regions. In this study, we used modern surface-based methods allowing estimation of multiple morphometric aspects of cortical structures. These metrics provide complementary information on the brain structure and allow to detect also minimal cortical alterations [21, 29–32]. We investigated several cortical metrics, including not only the well-known cortical thickness, volume and surface area, but also roughness and mean curvature. Roughness is a recently introduced metric calculated as the standard deviation of the cortical thickness, and an increase of this feature implies some degree of cortical atrophy [33]. Mean curvature values provide a quantitative measure of the cortical folding. Increased mean curvature indicates sharper cortical folds, which may reflect cortical atrophy or subcortical white matter atrophy [34]. In our study, rET patients showed increased roughness and mean curvature with normal thickness values in some fronto-temporal areas compared with HC and ET patients, suggesting that roughness and mean curvature may be more sensitive than classic metrics such as thickness in detecting cortical atrophy, a finding in agreement with a previous report [33]. A possible explanation for the higher cortical involvement we found in fronto-temporal areas in rET than in ET patients may be the cognitive status, as suggested by the lower cognitive scores in rET than controls and the significant correlations between imaging and cognitive data. More in detail, the COWAT score correlated with metrics of the parahippocampal and fusiform cortex, which is in line with previous studies [35, 36]. The parahippocampal, entorhinal and fusiform cortex, which showed increased roughness and mean curvature in rET patients, constitute a large part of the medial temporal lobe and play an important role in memory formation and language, since the parahippocampal gyrus provides a major source of input streams to the entorhinal cortex, and then directly into the hippocampus [36, 37]. The left pars opercularis, which showed significantly lower volume in rET compared to ET patients, is also involved in the language domain is part the interplay between temporal and frontal regions necessary for verbal fluency [38, 39]. Less clear is the correlation of COWAT with the paracentral cortex, which is mainly concerned with motor and sensory functions [40].

Differently from rET, we did not find differences in any cortical metric between ET and control subjects. This result is in line with the existing literature [41, 42] and may well reflect the lack of cognitive issues in “pure” ET patients. According to the second consensus on tremors, the presence of memory issues is considered as a soft neurological sign which makes the diagnosis change from ET to ET plus [2].

After demonstrating the presence of group differences between rET and ET patients in cortical metrics with the classic statistical univariate approach, we hypothesized that these two ET syndromes could be distinguished at the individual level using a machine learning approach based on structural metrics extracted from T1-weighted MR images. Recent advances in artificial intelligence technology applied on brain morphometric metrics have allowed to improve the classification of neurological disorders [16–21]. In the ET field, some authors demonstrated that machine learning models using cortical structural metrics (cortical thickness and roughness) yielded excellent performances in distinguishing ET from orthostatic tremor [21]. This previous study [21], however, did not include ET patients with rest tremor. In our study, the multivariate XGBoost classifier was able to discriminate between rET and ET patients with a good performance. Numerous models using different combinations of MRI structural metrics were compared and the model obtaining the best performance was based on cortical volume, showing mean AUC of 0.86 ± 0.11 in cross-validation analysis. Feature importance analysis identified the cortical volume in the left pars opercularis as the most informative feature for classification between the two ET syndromes. This result was in line with the statistical univariate approach which identified significantly lower cortical volume in this cortical region in rET than in ET patients.

These results, after validation in independent patient cohorts, may be useful to improve the differential diagnosis between these two tremor syndromes. The clinical classification into “ET” or “ET with rest tremor” is obviously guided by the presence or absence of tremor at rest. In these patients, however, the rest tremor may be not constant and often of low amplitude, and in our practice we also found some ET patients who had a rest tremor not clinically visible but detectable using surface electromyography. A recent study [9] showed in a large cohort of 200 ET patients that a significant percentage of patient changed diagnosis multiple times from ET to ET plus and vice versa over time, with rest tremor being the most unstable clinical feature. In this previous study [9], nearly 40% of patients who received a clinical diagnosis of “ET with rest tremor” were classified as “ET” in one or more follow-up visits and some of them back again to “ET with rest tremor” later on, providing evidence that an accurate clinical differential diagnosis between ET and rET may be challenging since rest tremor can fluctuate over time.

Our results should be interpreted within the context of some limitations. First, ET and rET patients had no post-mortem pathological examination, thus a misdiagnosis may have occurred in some cases; all patients, however, were diagnosed according to recent international diagnostic criteria [2], all rET patients had rest tremor confirmed by surface electromyography showing a synchronous contraction pattern, and all ET and rET patients had a normal DaTscan, thus ruling out Parkinson’s disease, which is the most common cause of rest tremor. Second, rET patients were slightly older and had lower education level than ET patients. However, we included these variables as covariates in all the analyses to minimize the possible bias in the results. Another possible limitation of our study, like most studies on essential tremor, is linked to the syndromic nature of ET and rET. According to the second consensus on the classification of tremor [2], ET and rET are indeed considered clinical syndromes rather than diseases, with multiple possible etiologies including genetic, acquired, and idiopathic disorders. This etiological heterogeneity may potentially lead to interindividual variability and thus reduce the significance of findings.

In conclusion, our study provides evidence of higher cortical atrophy in fronto-temporal regions in ET patients with rest tremor compared to those with classic ET, possibly reflecting higher cognitive deficits. A machine learning model combining cortical volumetric measures accurately discriminated between these two ET syndromes, helping the clinical differential diagnosis and further supporting the existence of different cortical involvement in ET patients with and without rest tremor.

## Supplementary Information

Below is the link to the electronic supplementary material.

Supplementary file1 (DOCX 16 kb)

Supplementary file2 (DOCX 15 kb)

Supplementary file3 (DOCX 14 kb)

Supplementary file4 (DOCX 34 kb)

Supplementary file5 (DOCX 35 kb)

## Data Availability

The data that support the results of this study are available from the corresponding author upon reasonable request.
