# Supplementary material for: Cortical involvement in essential tremor with and without rest tremor: a machine learning study
Source: J Neurol. 2023 May 5;270(8):4004–12. doi: 10.1007/s00415-023-11747-6 (PMC10344993; doi:10.1007/s00415-023-11747-6)
Supplement: Supplementary file 1 — Supplementary file1 (DOCX 16 kb) [file 415_2023_11747_MOESM1_ESM.docx]

**Supplementary Table 1.** Significant differences in cortical metrics among patients with essential tremor with and without rest tremor, and control subjects.

|  |  |  |  |  | **ANCOVA post-hoc** | |
| --- | --- | --- | --- | --- | --- | --- |
| **Regions** | **Lobe** | **rET** | **ET** | **CTRL** | **Adjusted p-value** | **Pairwise comparisons** |
| ***ROUGHNESS*** |  |  |  |  |  |  |
| lh_entorhinal | Temporal | 0.79 (0.02) | 0.78 (0.15) | 0.73 (0.01) | 0.02 | **rET>HC** |
| lh_parahippocampal | Temporal | 0.75 (0.02) | 0.69 (0.02) | 0.75 (0.01) | 0.01 | **rET> ET** |
| rh_parahippocampal | Temporal | 0.78 (0.02) | 0.71 (0.02) | 0.74 (0.01) | 0.03 | **rET> ET** |
| ***MEAN CURVATURE*** |  |  |  |  |  |  |
| lh_paracentral | Frontal | 0.109 (0.001) | 0.106 (0.001) | 0.102 (0.001) | 0.04 | **rET>HC** |
| rh_fusiform | Temporal | 0.134 (0.001) | 0.132 (0.001) | 0.129 (0.001) | 0.02 | **rET>HC** |
| lh_entorihinal | Temporal | 0.12 (0.003) | 0.11 (0.003) | 0.11 (0.002) | 0.04 | **rET> ET** |
| lh_parahippocampal | Temporal | 0.09 (0.001) | 0.08 (0.001) | 0.09 (0.001) | 0.02 | **rET> ET** |
| ***CORTICAL VOLUME*** |  |  |  |  |  |  |
| lh_pars opercularis | Frontal | 3813 (112) | 4269 (105.3) | 3973 (91.5) | 0.006 | **rET<ET** |

Abbreviations: rET = Essential Tremor with rest tremor; ET = essential tremor; HC = Healthy Controls,

Data are the mean values in each region, adjusted for covariates, expressed in mm for thickness and roughness, and in mm^-1^ for mean curvature. Data shown in brackets refer to standard error. Only significant differences are presented in the table. P values were adjusted for Bonferroni correction.

Statistical comparisons were performed using ANCOVA and post-hoc with age, sex and education as covariates; disease duration was also included as covariate in rET versus ET comparisons.

ANCOVA and post-hoc tests were also repeated including also Mini Mental State Examination score among covariates, and the results remained statistically significant in all metrics except for the right fusiform mean curvature.
