# Supplementary material for: Cortical involvement in essential tremor with and without rest tremor: a machine learning study
Source: J Neurol. 2023 May 5;270(8):4004–12. doi: 10.1007/s00415-023-11747-6 (PMC10344993; doi:10.1007/s00415-023-11747-6)
Supplement: Supplementary file 2 — Supplementary file2 (DOCX 15 kb) [file 415_2023_11747_MOESM2_ESM.docx]

**Supplementary Table 2.** Significant correlations between imaging and cognitive data in patients with essential tremor with rest tremor

| **Imaging data** | **COWAT/FAS raw** | **MMSE** |
| --- | --- | --- |
| **Lh parahippocampal roughness** | **-0.488 (0.04)** | NS |
| **Rh parahippocampal roughness** | **-0.754 (0.0004)** | -0.459 (0.02) |
| **Lh entorhinal roughness** | NS | -0.487 (0.02) |
| **Lh entorhinal mean curvature** | -0.493 (0.04) | NS |
| **Lh paracentral mean curvature** | **-0.519 (0.03)** | NS |
| **Rh fusiform mean curvature** | **-0.534 (0.02)** | NS |

Abbreviations: Lh = left; Rh = right. Results are expressed as Spearman’s rho correlation coefficient (p value). Possible correlations were investigated between cognitive scores and brain metrics significantly different between rET patients and other groups (ET or controls). Values in bold remain statistically significant also including age and education level as covariates in the correlation analysis.
