# Supplementary material for: Cortical involvement in essential tremor with and without rest tremor: a machine learning study
Source: J Neurol. 2023 May 5;270(8):4004–12. doi: 10.1007/s00415-023-11747-6 (PMC10344993; doi:10.1007/s00415-023-11747-6)
Supplement: Supplementary file 3 — Supplementary file3 (DOCX 14 kb) [file 415_2023_11747_MOESM3_ESM.docx]

**Supplementary Table 3.** Partial correlations between imaging and rest tremor features in patients with essential tremor with rest tremor

|  | **Amplitude** | **Phase** | **Frequency** |
| --- | --- | --- | --- |
| **Lh parahippocampal roughness** | -0.021 (NS) | -0.013 (NS) | 0.029 (NS) |
| **Rh parahippocampal roughness** | 0.182 (NS) | 0.246 (NS) | 0.278 (NS) |
| **Lh entorhinal roughness** | 0.270 (NS) | 0.057 (NS) | -0.007 (NS) |
| **Lh entorhinal mean curvature** | -0.308 (NS) | 0.025 (NS) | -0.059 (NS) |
| **Lh paracentral mean curvature** | 0.146 (NS) | 0.065 (NS) | 0.128 (NS) |
| **Rh fusiform mean curvature** | -0.176 (NS) | 0.105 (NS) | -0.179 (NS) |
| **Lh parahippocampal mean curvature** | 0.293 (NS) | 0.127 (NS) | -0.129 (NS) |

Abbreviations: Lh = left; Rh = right. Results are expressed as Spearman’s rho correlation coefficient (p value). Possible correlations were investigated between rest tremor electrophysiological features and brain metrics significantly different between rET patients and other groups (ET or controls). Age and education level were included as covariates in the correlation analysis.
