# Supplementary material for: Cortical involvement in essential tremor with and without rest tremor: a machine learning study
Source: J Neurol. 2023 May 5;270(8):4004–12. doi: 10.1007/s00415-023-11747-6 (PMC10344993; doi:10.1007/s00415-023-11747-6)
Supplement: Supplementary file 4 — Supplementary file4 (DOCX 34 kb) [file 415_2023_11747_MOESM4_ESM.docx]

**Supplementary Table 4:** Classification performances of all XGBOOST models in distinguishing between essential tremor patients with and without rest tremor.

MODEL A THICKNESS

MODEL B CORTICAL VOLUME

MODEL C SURFACE AREA

MODEL D SUBCORTICAL VOLUME

MODEL E MEAN CURVATURE

MODEL F ROUGHNESS

|  | **rET vs ET** | | | | | |
| --- | --- | --- | --- | --- | --- | --- |
|  | **A** | **B** | **C** | **D** | **E** | **F** |
| **ALL FEATURES** | AUC : 0.602 (0.14) | **AUC : 0.726 (0.171)** | AUC : 0.539 (0.147) | AUC : 0.536 (0.089) | AUC : 0.676 (0.162) | AUC : 0.5  (0.0) |
|  | ACC : 0.576 (0.121) | **ACC : 0.675 (0.128)** | ACC : 0.529 (0.141) | ACC : 0.548 (0.102) | ACC : 0.614 (0.15) | ACC : 0.523 (0.019) |
|  | SENS : 0.48 (0.19) | **SENS : 0.627 (0.184)** | SENS : 0.453 (0.203) | SENS : 0.52 (0.207) | SENS : 0.567 (0.254) | SENS :  0.0 (0.0) |
|  | SPEC : 0.661 (0.182) | **SPEC : 0.723 (0.171)** | SPEC : 0.6 (0.162) | SPEC : 0.574 (0.158) | SPEC : 0.658 (0.178) | SPEC : 1.0  (0.0) |
| **FEATURE SELECTION** | AUC : 0.659 (0.141) | **AUC : 0.865 (0.114)** | AUC : 0.721 (0.122) | AUC : 0.732 (0.119) | AUC : 0.767 (0.124) | AUC : 0.5  (0.0) |
|  | ACC : 0.625 (0.116) | **ACC : 0.809 (0.119)** | ACC : 0.721 (0.125) | ACC : 0.704 (0.105) | ACC : 0.711 (0.144) | ACC : 0.523 (0.019) |
|  | SENS : 0.54 (0.151) | **SENS : 0.78 (0.187)** | SENS : 0.733 (0.176) | SENS : 0.72 (0.154) | SENS : 0.707 (0.251) | SENS :  0.0 (0.0) |
|  | SPEC : 0.702 (0.184) | **SPEC : 0.836 (0.13)** | SPEC : 0.709 (0.218) | SPEC : 0.688 (0.168) | SPEC : 0.712 (0.133) | SPEC : 1.0  (0.0) |
| **N. OF FEATURES** | 63 | **9** | 1 | 5 | 4 | 1 |
|  |  |  |  |  |  |  |
|  | **AB** | **AC** | **AD** | **BC** | **BD** | **CD** |
| **ALL FEATURES** | AUC : 0.656 (0.167) | AUC : 0.555 (0.153) | AUC : 0.622 (0.155) | AUC : 0.716 (0.143) | AUC : 0.738 (0.177) | AUC : 0.483 (0.143) |
|  | ACC : 0.609 (0.144) | ACC : 0.565 (0.129) | ACC : 0.583 (0.111) | ACC : 0.685 (0.117) | ACC : 0.656 (0.116) | ACC : 0.479 (0.106) |
|  | SENS : 0.527 (0.229) | SENS : 0.44 (0.169) | SENS : 0.44 (0.148) | SENS : 0.613 (0.168) | SENS : 0.56 (0.176) | SENS : 0.427 (0.189) |
|  | SPEC : 0.685 (0.161) | SPEC : 0.679 (0.204) | SPEC : 0.711 (0.203) | SPEC : 0.753 (0.165) | SPEC : 0.749 (0.205) | SPEC : 0.53 (0.172) |
| **FEATURE SELECTION** | AUC : 0.82 (0.125) | AUC : 0.689 (0.169) | AUC : 0.663 (0.169) | AUC : 0.763 (0.168) | AUC : 0.759 (0.177) | AUC : 0.759 (0.138) |
|  | ACC : 0.762 (0.111) | ACC : 0.64 (0.142) | ACC : 0.643 (0.144) | ACC : 0.708 (0.136) | ACC : 0.72 (0.142) | ACC : 0.701 (0.106) |
|  | SENS : 0.713 (0.167) | SENS : 0.547 (0.219) | SENS : 0.56 (0.205) | SENS : 0.66 (0.203) | SENS : 0.66 (0.208) | SENS : 0.707 (0.172) |
|  | SPEC : 0.811 (0.147) | SPEC : 0.73 (0.18) | SPEC : 0.718 (0.183) | SPEC : 0.756 (0.187) | SPEC : 0.778 (0.177) | SPEC : 0.696 (0.147) |
| **N. OF FEATURES** | 16 | 34 | 39 | 69 | 55 | 16 |
|  |  |  |  |  |  |  |
|  | **AE** | **BE** | **CE** | **DE** | **AF** | **BF** |
| **ALL FEATURES** | AUC : 0.59 (0.141) | AUC : 0.699 (0.165) | AUC : 0.622 (0.148) | AUC : 0.645 (0.144) | AUC : 0.598 (0.14) | AUC : 0.657 (0.161) |
|  | ACC : 0.563 (0.118) | ACC : 0.659 (0.137) | ACC : 0.566 (0.137) | ACC : 0.617 (0.152) | ACC : 0.579 (0.127) | ACC : 0.605 (0.123) |
|  | SENS : 0.527 (0.234) | SENS : 0.6 (0.2) | SENS : 0.547 (0.197) | SENS : 0.54 (0.184) | SENS : 0.467 (0.194) | SENS : 0.533 (0.216) |
|  | SPEC : 0.594 (0.183) | SPEC : 0.715 (0.205) | SPEC : 0.584 (0.162) | SPEC : 0.688 (0.209) | SPEC : 0.682 (0.176) | SPEC : 0.675 (0.17) |
| **FEATURE SELECTION** | AUC : 0.759 (0.154) | AUC : 0.784 (0.147) | AUC : 0.777 (0.12) | AUC : 0.755 (0.101) | AUC : 0.682 (0.146) | AUC : 0.797 (0.119) |
|  | ACC : 0.716 (0.138) | ACC : 0.743 (0.126) | ACC : 0.736 (0.12) | ACC : 0.686 (0.088) | ACC : 0.651 (0.102) | ACC : 0.755 (0.131) |
|  | SENS : 0.72 (0.198) | SENS : 0.727 (0.199) | SENS : 0.727 (0.215) | SENS : 0.64 (0.168) | SENS : 0.56 (0.169) | SENS : 0.667 (0.216) |
|  | SPEC : 0.711 (0.139) | SPEC : 0.759 (0.173) | SPEC : 0.741 (0.169) | SPEC : 0.73 (0.16) | SPEC : 0.732 (0.15) | SPEC : 0.84 (0.136) |
| **N. OF FEATURES** | 11 | 9 | 5 | 14 | 38 | 3 |
|  |  |  |  |  |  |  |
|  | **CF** | **DF** | **EF** | **ABC** | **ABD** | **ACD** |
| **ALL FEATURES** | AUC : 0.5  (0.0) | AUC : 0.5  (0.0) | AUC : 0.566 (0.12) | AUC : 0.612 (0.181) | AUC : 0.62 (0.155) | AUC : 0.497 (0.184) |
|  | ACC : 0.523 (0.019) | ACC : 0.523 (0.019) | ACC : 0.548 (0.127) | ACC : 0.574 (0.138) | ACC : 0.584 (0.115) | ACC : 0.467 (0.132) |
|  | SENS : 0.0  (0.0) | SENS : 0.0  (0.0) | SENS : 0.487 (0.235) | SENS : 0.5 (0.216) | SENS : 0.513 (0.182) | SENS : 0.36 (0.209) |
|  | SPEC : 1.0  (0.0) | SPEC : 1.0  (0.0) | SPEC : 0.605 (0.185) | SPEC : 0.641 (0.168) | SPEC : 0.65 (0.174) | SPEC : 0.563 (0.213) |
| **FEATURE SELECTION** | AUC : 0.5  (0.0) | AUC : 0.5  (0.0) | AUC : 0.74 (0.119) | AUC : 0.698 (0.148) | AUC : 0.747 (0.112) | AUC : 0.729 (0.122) |
|  | ACC : 0.523 (0.019) | ACC : 0.523 (0.019) | ACC : 0.676 (0.126) | ACC : 0.705 (0.122) | ACC : 0.696 (0.122) | ACC : 0.693 (0.113) |
|  | SENS : 0.0  (0.0) | SENS : 0.0  (0.0) | SENS : 0.607 (0.278) | SENS : 0.687 (0.151) | SENS : 0.7 (0.163) | SENS : 0.573 (0.189) |
|  | SPEC : 1.0  (0.0) | SPEC : 1.0  (0.0) | SPEC : 0.739 (0.147) | SPEC : 0.727 (0.176) | SPEC : 0.695 (0.167) | SPEC : 0.8 (0.168) |
| **N. OF FEATURES** | 1 | 1 | 48 | 1 | 3 | 1 |
|  |  |  |  |  |  |  |
|  | **BCD** | **ABE** | **ABF** | **ACE** | **ACF** | **ADE** |
| **ALL FEATURES** | AUC : 0.683 (0.127) | AUC : 0.628 (0.146) | AUC : 0.661 (0.187) | AUC : 0.586 (0.171) | AUC : 0.588 (0.176) | AUC : 0.582 (0.138) |
|  | ACC : 0.638 (0.106) | ACC : 0.614 (0.124) | ACC : 0.586 (0.123) | ACC : 0.547 (0.121) | ACC : 0.526 (0.124) | ACC : 0.584 (0.107) |
|  | SENS : 0.54 (0.19) | SENS : 0.547 (0.18) | SENS : 0.48 (0.218) | SENS : 0.48 (0.196) | SENS : 0.44 (0.205) | SENS : 0.487 (0.221) |
|  | SPEC : 0.73 (0.152) | SPEC : 0.675 (0.169) | SPEC : 0.686 (0.179) | SPEC : 0.609 (0.176) | SPEC : 0.605 (0.221) | SPEC : 0.674 (0.211) |
| **FEATURE SELECTION** | AUC : 0.828 (0.113) | AUC : 0.767 (0.136) | AUC : 0.686 (0.154) | AUC : 0.729 (0.137) | AUC : 0.659 (0.171) | AUC : 0.712 (0.13) |
|  | ACC : 0.765 (0.1) | ACC : 0.686 (0.127) | ACC : 0.642 (0.138) | ACC : 0.689 (0.127) | ACC : 0.621 (0.129) | ACC : 0.705 (0.108) |
|  | SENS : 0.733 (0.189) | SENS : 0.653 (0.188) | SENS : 0.593 (0.236) | SENS : 0.68 (0.182) | SENS : 0.533 (0.176) | SENS : 0.653 (0.169) |
|  | SPEC : 0.796 (0.157) | SPEC : 0.719 (0.13) | SPEC : 0.689 (0.171) | SPEC : 0.695 (0.183) | SPEC : 0.702 (0.192) | SPEC : 0.754 (0.139) |
| **N. OF FEATURES** | 6 | 3 | 155 | 1 | 99 | 2 |
|  |  |  |  |  |  |  |
|  | **ADF** | **AEF** | **BEF** | **CEF** | **DEF** | **BCE** |
| **ALL FEATURES** | AUC : 0.589 (0.124) | AUC : 0.5  (0.0) | AUC : 0.657 (0.164) | AUC : 0.5  (0.0) | AUC : 0.5  (0.0) | AUC : 0.68 (0.179) |
|  | ACC : 0.548 (0.124) | ACC : 0.523 (0.019) | ACC : 0.621 (0.144) | ACC : 0.523 (0.019) | ACC : 0.523 (0.019) | ACC : 0.619 (0.149) |
|  | SENS : 0.427 (0.189) | SENS : 0.0  (0.0) | SENS : 0.553 (0.209) | SENS : 0.0  (0.0) | SENS : 0.0 (  0.0) | SENS : 0.56 (0.21) |
|  | SPEC : 0.655 (0.207) | SPEC : 1.0  (0.0) | SPEC : 0.684 (0.159) | SPEC : 1.0  (0.0) | SPEC : 1.0  (0.0) | SPEC : 0.679 (0.199) |
| **FEATURE SELECTION** | AUC : 0.711 (0.163) | AUC : 0.5  (0.0) | AUC : 0.827 (0.134) | AUC : 0.5  (0.0) | AUC : 0.5  (0.0) | AUC : 0.743 (0.123) |
|  | ACC : 0.667 (0.13) | ACC : 0.523 (0.019) | ACC : 0.749 (0.102) | ACC : 0.523 (0.019) | ACC : 0.523 (0.019) | ACC : 0.685 (0.122) |
|  | SENS : 0.573 (0.183) | SENS : 0.0  (0.0) | SENS : 0.753 (0.177) | SENS : 0.0  (0.0) | SENS : 0.0  (0.0) | SENS : 0.667 (0.183) |
|  | SPEC : 0.751 (0.161) | SPEC : 1.0  (0.0) | SPEC : 0.75 (0.162) | SPEC : 1.0  (0.0) | SPEC : 1.0  (0.0) | SPEC : 0.702 (0.169) |
| **N. OF FEATURES** | 46 | 1 | 9 | 1 | 1 | 14 |
|  |  |  |  |  |  |  |
|  | **BCF** | **BDE** | **BDF** | **CDE** | **CDF** | **ABCD** |
| **ALL FEATURES** | AUC : 0.62 (0.165) | AUC : 0.711 (0.174) | AUC : 0.689 (0.165) | AUC : 0.597 (0.166) | AUC : 0.572 (0.164) | AUC : 0.672 (0.143) |
|  | ACC : 0.59 (0.12) | ACC : 0.658 (0.137) | ACC : 0.637 (0.146) | ACC : 0.591 (0.104) | ACC : 0.542 (0.115) | ACC : 0.599 (0.123) |
|  | SENS : 0.527 (0.174) | SENS : 0.607 (0.182) | SENS : 0.527 (0.248) | SENS : 0.54 (0.172) | SENS : 0.427 (0.231) | SENS : 0.493 (0.213) |
|  | SPEC : 0.651 (0.196) | SPEC : 0.709 (0.186) | SPEC : 0.742 (0.19) | SPEC : 0.639 (0.173) | SPEC : 0.65 (0.216) | SPEC : 0.698 (0.172) |
| **FEATURE SELECTION** | AUC : 0.826 (0.112) | AUC : 0.809 (0.138) | AUC : 0.773 (0.119) | AUC : 0.769 (0.142) | AUC : 0.673 (0.158) | AUC : 0.69 (0.18) |
|  | ACC : 0.733 (0.12) | ACC : 0.748 (0.141) | ACC : 0.716 (0.115) | ACC : 0.712 (0.106) | ACC : 0.633 (0.134) | ACC : 0.664 (0.13) |
|  | SENS : 0.66 (0.208) | SENS : 0.713 (0.186) | SENS : 0.687 (0.178) | SENS : 0.727 (0.162) | SENS : 0.553 (0.204) | SENS : 0.613 (0.234) |
|  | SPEC : 0.803 (0.14) | SPEC : 0.782 (0.164) | SPEC : 0.745 (0.145) | SPEC : 0.695 (0.13) | SPEC : 0.708 (0.189) | SPEC : 0.711 (0.152) |
| **N. OF FEATURES** | 7 | 14 | 38 | 2 | 88 | 60 |
|  |  |  |  |  |  |  |
|  | **ABCE** | **ABCF** | **ABEF** | **ACDE** | **ACDF** | **BCDE** |
| **ALL FEATURES** | AUC : 0.667 (0.161) | AUC : 0.651 (0.162) | AUC : 0.64 (0.151) | AUC : 0.605 (0.177) | AUC : 0.5  (0.0) | AUC : 0.679 (0.162) |
|  | ACC : 0.65 (0.135) | ACC : 0.596 (0.103) | ACC : 0.574 (0.132) | ACC : 0.591 (0.129) | ACC : 0.523 (0.019) | ACC : 0.629 (0.161) |
|  | SENS : 0.62 (0.208) | SENS : 0.547 (0.208) | SENS : 0.52 (0.196) | SENS : 0.473 (0.181) | SENS : 0.0  (0.0) | SENS : 0.56 (0.221) |
|  | SPEC : 0.68 (0.213) | SPEC : 0.644 (0.137) | SPEC : 0.623 (0.197) | SPEC : 0.697 (0.2) | SPEC : 1.0  (0.0) | SPEC : 0.696 (0.207) |
| **FEATURE SELECTION** | AUC : 0.777 (0.132) | AUC : 0.8  (0.125) | AUC : 0.698 (0.148) | AUC : 0.616 (0.159) | AUC : 0.5  (0.0) | AUC : 0.736 (0.137) |
|  | ACC : 0.709 (0.122) | ACC : 0.749 (0.134) | ACC : 0.705 (0.122) | ACC : 0.615 (0.11) | ACC : 0.523 (0.019) | ACC : 0.726 (0.106) |
|  | SENS : 0.727 (0.162) | SENS : 0.687 (0.196) | SENS : 0.687 (0.151) | SENS : 0.54 (0.172) | SENS : 0.0  (0.0) | SENS : 0.72 (0.168) |
|  | SPEC : 0.698 (0.182) | SPEC : 0.81 (0.161) | SPEC : 0.727 (0.176) | SPEC : 0.685 (0.183) | SPEC : 1.0  (0.0) | SPEC : 0.732 (0.152) |
| **N. OF FEATURES** | 3 | 4 | 1 | 77 | 1 | 7 |
|  |  |  |  |  |  |  |
|  | **BCDF** | **BCEF** | **BDEF** | **CDEF** | **ABDF** | **ADEF** |
| **ALL FEATURES** | AUC : 0.657 (0.156) | AUC : 0.664 (0.173) | AUC : 0.701 (0.142) | AUC : 0.5  (0.0) | AUC : 0.639 (0.164) | AUC : 0.5  (0.0) |
|  | ACC : 0.622 (0.119) | ACC : 0.635 (0.137) | ACC : 0.659 (0.121) | ACC : 0.523 (0.019) | ACC : 0.593 (0.132) | ACC : 0.523 (0.019) |
|  | SENS : 0.573 (0.195) | SENS : 0.593 (0.211) | SENS : 0.607 (0.194) | SENS : 0.0  (0.0) | SENS : 0.52 (0.228) | SENS : 0.0  (0.0) |
|  | SPEC : 0.67 (0.196) | SPEC : 0.678 (0.209) | SPEC : 0.71 (0.196) | SPEC : 1.0  (0.0) | SPEC : 0.661 (0.195) | SPEC : 1.0  (0.0) |
| **FEATURE SELECTION** | AUC : 0.789 (0.13) | AUC : 0.764 (0.141) | AUC : 0.833 (0.105) | AUC : 0.5  (0.0) | AUC : 0.698 (0.148) | AUC : 0.5  (0.0) |
|  | ACC : 0.733 (0.139) | ACC : 0.734 (0.125) | ACC : 0.751 (0.147) | ACC : 0.523 (0.019) | ACC : 0.705 (0.122) | ACC : 0.523 (0.019) |
|  | SENS : 0.687 (0.202) | SENS : 0.68 (0.21) | SENS : 0.667 (0.2) | SENS : 0.0  (0.0) | SENS : 0.687 (0.151) | SENS : 0.0  (0.0) |
|  | SPEC : 0.78 (0.186) | SPEC : 0.784 (0.133) | SPEC : 0.831 (0.17) | SPEC : 1.0  (0.0) | SPEC : 0.727 (0.176) | SPEC : 1.0  (0.0) |
| **N. OF FEATURES** | 3 | 69 | 4 | 1 | 1 | 1 |
|  |  |  |  |  |  |  |
|  | **ACFE** | **ABCDE** | **ABCDF** | **ACDEF** | **BCDEF** | **ABDEF** |
| **ALL FEATURES** | AUC : 0.5  (0.0) | AUC : 0.668 (0.166) | AUC : 0.674 (0.157) | AUC : 0.5  (0.0) | AUC : 0.632 (0.188) | AUC : 0.653 (0.165) |
|  | ACC : 0.523 (0.019) | ACC : 0.631 (0.152) | ACC : 0.583 (0.119) | ACC : 0.523 (0.019) | ACC : 0.6  (0.137) | ACC : 0.593 (0.152) |
|  | SENS : 0.0  (0.0) | SENS : 0.587 (0.211) | SENS : 0.46 (0.19) | SENS : 0.0  (0.0) | SENS : 0.52 (0.223) | SENS : 0.487 (0.221) |
|  | SPEC : 1.0  (0.0) | SPEC : 0.673 (0.2) | SPEC : 0.697 (0.177) | SPEC : 1.0  (0.0) | SPEC : 0.676 (0.19) | SPEC : 0.692 (0.2) |
| **FEATURE SELECTION** | AUC : 0.5  (0.0) | AUC : 0.706 (0.143) | AUC : 0.772 (0.15) | AUC : 0.5  (0.0) | AUC : 0.698 (0.148) | AUC : 0.791 (0.175) |
|  | ACC : 0.523 (0.019) | ACC : 0.715 (0.132) | ACC : 0.73 (0.124) | ACC : 0.523 (0.019) | ACC : 0.705 (0.122) | ACC : 0.729 (0.14) |
|  | SENS : 0.0  (0.0) | SENS : 0.713 (0.153) | SENS : 0.68 (0.194) | SENS : 0.0  (0.0) | SENS : 0.687 (0.151) | SENS : 0.647 (0.218) |
|  | SPEC : 1.0  (0.0) | SPEC : 0.721 (0.179) | SPEC : 0.781 (0.179) | SPEC : 1.0  (0.0) | SPEC : 0.727 (0.176) | SPEC : 0.81 (0.168) |
| **N. OF FEATURES** | 1 | 2 | 56 | 1 | 1 | 58 |
|  |  |  |  |  |  |  |
|  | **ABCEF** | **ABCDEF** |  |  |  |  |
| **ALL FEATURES** | AUC : 0.636 (0.17) | AUC : 0.649 (0.167) |  |  |  |  |
|  | ACC : 0.584 (0.138) | ACC : 0.587 (0.123) |  |  |  |  |
|  | SENS : 0.533 (0.189) | SENS : 0.48 (0.212) |  |  |  |  |
|  | SPEC : 0.632 (0.186) | SPEC : 0.687 (0.177) |  |  |  |  |
| **FEATURE SELECTION** | AUC : 0.695 (0.157) | AUC : 0.73 (0.146) |  |  |  |  |
|  | ACC : 0.683 (0.119) | ACC : 0.7  (0.136) |  |  |  |  |
|  | SENS : 0.673 (0.16) | SENS : 0.68 (0.188) |  |  |  |  |
|  | SPEC : 0.697 (0.182) | SPEC : 0.719 (0.172) |  |  |  |  |
| **N. OF FEATURES** | 1 | 36 |  |  |  |  |

Abbreviations: rET = Essential tremor with rest tremor; ET = Essential tremor; AUC = Area Under the Curve. The best model is highlighted in bold.
