# Supplementary material for: Cortical involvement in essential tremor with and without rest tremor: a machine learning study
Source: J Neurol. 2023 May 5;270(8):4004–12. doi: 10.1007/s00415-023-11747-6 (PMC10344993; doi:10.1007/s00415-023-11747-6)
Supplement: Supplementary file 5 — Supplementary file5 (DOCX 35 kb) [file 415_2023_11747_MOESM5_ESM.docx]

**Supplementary Table 5:** Classification performances of all XGBOOST models in distinguishing essential tremor patients with rest tremor from control subjects.

MODEL A THICKNESS

MODEL B CORTICAL VOLUME

MODEL C SURFACE AREA

MODEL D SUBCORTICAL VOLUME

MODEL E MEAN CURVATURE

MODEL F ROUGHNESS

|  | **rET vs Control subjects** | | | | | |
| --- | --- | --- | --- | --- | --- | --- |
|  | **A** | **B** | **C** | **D** | **E** | **F** |
| **ALL FEATURES** | AUC :0.371 (0.12) | AUC :0.534 (0.135) | AUC :0.361 (0.128) | AUC :0.533 (0.152) | AUC :0.561 (0.112) | **AUC :0.664 (0.123)** |
|  | ACC :0.525 (0.083) | ACC :0.547 (0.108) | ACC :0.568 (0.057) | ACC :0.536 (0.129) | ACC :0.587 (0.078) | **ACC :0.645 (0.105)** |
|  | SENS :0.067 (0.094) | SENS :0.333 (0.149) | SENS :  0.047 (0.1) | SENS :0.28 (0.234) | SENS :0.173 (0.153) | **SENS :0.433 (0.17)** |
|  | SPEC : 0.831 (0.13) | SPEC : 0.689 (0.154) | SPEC :  0.916 (0.085) | SPEC : 0.707 (0.16) | SPEC : 0.862 (0.134) | **SPEC : 0.787 (0.121)** |
| **FEATURE SELECTION** | AUC : 0.66 (0.077) | AUC : 0.727 (0.125) | AUC : 0.596 (0.122) | AUC : 0.544 (0.128) | AUC : 0.689 (0.102) | **AUC : 0.85 (0.091)** |
|  | ACC : 0.661 (0.068) | ACC : 0.725 (0.126) | ACC : 0.611 (0.062) | ACC : 0.651 (0.076) | ACC : 0.653 (0.075) | **ACC : 0.813 (0.088)** |
|  | SENS : 0.407 (0.164) | SENS : 0.54 (0.217) | SENS : 0.12 (0.138) | SENS : 0.22 (0.147) | SENS : 0.253 (0.201) | **SENS : 0.693 (0.198)** |
|  | SPEC : 0.831 (0.095) | SPEC : 0.849 (0.163) | SPEC : 0.938 (0.084) | SPEC : 0.938 (0.084) | SPEC : 0.92 (0.097) | **SPEC : 0.893 (0.111)** |
| **N. OF FEATURES** | 2 | 7 | 6 | 2 | 3 | 14 |
|  |  |  |  |  |  |  |
|  | **AB** | **AC** | **AD** | **BC** | **BD** | **CD** |
| **ALL FEATURES** | AUC :0.484 (0.115) | AUC :0.339 (0.102) | AUC :0.36 (0.09) | AUC :0.471 (0.143) | AUC :0.455 (0.126) | AUC :0.387 (0.136) |
|  | ACC :0.52 (0.1) | ACC :0.485 (0.091) | ACC :0.48 (0.092) | ACC :0.573 (0.078) | ACC :0.528 (0.086) | ACC :0.485 (0.091) |
|  | SENS :0.28 (0.175) | SENS :0.14 (0.112) | SENS :0.213 (0.153) | SENS :0.133 (0.125) | SENS :0.127 (0.098) | SENS :0.213 (0.153) |
|  | SPEC : 0.68 (0.138) | SPEC : 0.716 (0.157) | SPEC : 0.658 (0.196) | SPEC : 0.867 (0.133) | SPEC : 0.796 (0.162) | SPEC : 0.667 (0.118) |
| **FEATURE SELECTION** | AUC : 0.796 (0.101) | AUC : 0.649 (0.126) | AUC : 0.659 (0.11) | AUC : 0.66 (0.143) | AUC : 0.66 (0.143) | AUC : 0.661 (0.137) |
|  | ACC : 0.749 (0.097) | ACC : 0.627 (0.115) | ACC : 0.648 (0.091) | ACC : 0.685 (0.112) | ACC : 0.685 (0.112) | ACC : 0.656 (0.109) |
|  | SENS : 0.633 (0.176) | SENS : 0.427 (0.2) | SENS : 0.46 (0.178) | SENS : 0.36 (0.174) | SENS : 0.36 (0.174) | SENS : 0.473 (0.187) |
|  | SPEC : 0.827 (0.114) | SPEC : 0.76 (0.136) | SPEC : 0.773 (0.185) | SPEC : 0.902 (0.127) | SPEC : 0.902 (0.127) | SPEC : 0.778 (0.113) |
| **N. OF FEATURES** | 8 | 10 | 6 | 2 | 2 | 7 |
|  |  |  |  |  |  |  |
|  | **AE** | **BE** | **CE** | **DE** | **AF** | **BF** |
| **ALL FEATURES** | AUC :0.488 (0.124) | AUC :0.529 (0.087) | AUC :0.468 (0.129) | AUC :0.438 (0.131) | AUC :0.564 (0.128) | AUC :0.604 (0.096) |
|  | ACC :0.547 (0.082) | ACC :0.579 (0.079) | ACC :0.555 (0.067) | ACC :0.571 (0.091) | ACC :0.571 (0.1) | ACC :0.573 (0.09) |
|  | SENS :0.08 (0.107) | SENS :0.167 (0.133) | SENS :0.107 (0.114) | SENS :0.12 (0.1) | SENS :0.307 (0.174) | SENS :0.313 (0.165) |
|  | SPEC : 0.858 (0.107) | SPEC : 0.853 (0.125) | SPEC : 0.853 (0.132) | SPEC : 0.871 (0.136) | SPEC : 0.747 (0.135) | SPEC : 0.747 (0.124) |
| **FEATURE SELECTION** | AUC : 0.734 (0.119) | AUC : 0.766 (0.143) | AUC : 0.687 (0.122) | AUC : 0.696 (0.091) | AUC : 0.814 (0.082) | AUC : 0.793 (0.089) |
|  | ACC : 0.648 (0.074) | ACC : 0.688 (0.096) | ACC : 0.677 (0.07) | ACC : 0.659 (0.066) | ACC : 0.757 (0.092) | ACC : 0.723 (0.07) |
|  | SENS : 0.187 (0.151) | SENS : 0.307 (0.204) | SENS : 0.22 (0.154) | SENS : 0.193 (0.154) | SENS : 0.627 (0.202) | SENS : 0.62 (0.16) |
|  | SPEC : 0.956 (0.07) | SPEC : 0.942 (0.071) | SPEC : 0.982 (0.041) | SPEC : 0.969 (0.067) | SPEC : 0.844 (0.113) | SPEC : 0.791 (0.096) |
| **N. OF FEATURES** | 6 | 6 | 4 | 4 | 6 | 5 |
|  |  |  |  |  |  |  |
|  | **CF** | **DF** | **EF** | **ABC** | **ABD** | **ACD** |
| **ALL FEATURES** | AUC :0.613 (0.161) | AUC :0.605 (0.127) | AUC :0.553 (0.119) | AUC :0.442 (0.095) | AUC :0.473 (0.1) | AUC :0.309 (0.103) |
|  | ACC :0.595 (0.118) | ACC :0.619 (0.123) | ACC :0.571 (0.105) | ACC :0.517 (0.1) | ACC :0.563 (0.08) | ACC :0.459 (0.083) |
|  | SENS :0.32 (0.235) | SENS :0.433 (0.163) | SENS :0.32 (0.2) | SENS :0.227 (0.155) | SENS :0.107 (0.104) | SENS :0.133 (0.105) |
|  | SPEC : 0.778 (0.133) | SPEC : 0.742 (0.146) | SPEC : 0.738 (0.125) | SPEC : 0.711 (0.154) | SPEC : 0.867 (0.118) | SPEC : 0.676 (0.154) |
| **FEATURE SELECTION** | AUC : 0.81 (0.123) | AUC : 0.82 (0.102) | AUC : 0.802 (0.089) | AUC : 0.721 (0.084) | AUC : 0.699 (0.109) | AUC : 0.661 (0.121) |
|  | ACC : 0.741 (0.118) | ACC : 0.744 (0.099) | ACC : 0.76 (0.098) | ACC : 0.723 (0.084) | ACC : 0.677 (0.094) | ACC : 0.621 (0.113) |
|  | SENS : 0.6 (0.262) | SENS : 0.647 (0.218) | SENS : 0.653 (0.21) | SENS : 0.633 (0.211) | SENS : 0.36 (0.198) | SENS : 0.413 (0.206) |
|  | SPEC : 0.836 (0.134) | SPEC : 0.809 (0.097) | SPEC : 0.831 (0.118) | SPEC : 0.782 (0.124) | SPEC : 0.889 (0.113) | SPEC : 0.76 (0.132) |
| **N. OF FEATURES** | 5 | 6 | 11 | 3 | 2 | 7 |
|  |  |  |  |  |  |  |
|  | **BCD** | **ABE** | **ABF** | **ACE** | **ACF** | **ADE** |
| **ALL FEATURES** | AUC :0.415 (0.123) | AUC :0.479 (0.108) | AUC :0.543 (0.133) | AUC :0.324 (0.098) | AUC :0.504 (0.152) | AUC :0.432 (0.133) |
|  | ACC :0.507 (0.098) | ACC :0.509 (0.082) | ACC :0.579 (0.096) | ACC :0.459 (0.089) | ACC :0.528 (0.108) | ACC :0.531 (0.091) |
|  | SENS :0.227 (0.148) | SENS :0.26 (0.125) | SENS :0.313 (0.19) | SENS :0.12 (0.138) | SENS :0.213 (0.153) | SENS :0.073 (0.083) |
|  | SPEC : 0.693 (0.138) | SPEC : 0.676 (0.121) | SPEC : 0.756 (0.113) | SPEC : 0.684 (0.132) | SPEC : 0.738 (0.147) | SPEC : 0.836 (0.134) |
| **FEATURE SELECTION** | AUC : 0.686 (0.141) | AUC : 0.752 (0.104) | AUC : 0.791 (0.103) | AUC : 0.597 (0.133) | AUC : 0.716 (0.085) | AUC : 0.724 (0.099) |
|  | ACC : 0.664 (0.102) | ACC : 0.733 (0.094) | ACC : 0.747 (0.098) | ACC : 0.627 (0.098) | ACC : 0.656 (0.067) | ACC : 0.643 (0.056) |
|  | SENS : 0.36 (0.154) | SENS : 0.567 (0.211) | SENS : 0.553 (0.198) | SENS : 0.48 (0.202) | SENS : 0.513 (0.182) | SENS : 0.2 (0.141) |
|  | SPEC : 0.867 (0.13) | SPEC : 0.844 (0.109) | SPEC : 0.876 (0.079) | SPEC : 0.724 (0.134) | SPEC : 0.751 (0.09) | SPEC : 0.938 (0.084) |
| **N. OF FEATURES** | 2 | 12 | 4 | 1 | 9 | 5 |
|  |  |  |  |  |  |  |
|  | **ADF** | **AEF** | **BEF** | **CEF** | **DEF** | **BCE** |
| **ALL FEATURES** | AUC :0.531 (0.139) | AUC :0.536 (0.135) | AUC :0.586 (0.102) | AUC :0.515 (0.109) | AUC :0.539 (0.134) | AUC :0.458 (0.122) |
|  | ACC :0.544 (0.12) | ACC :0.552 (0.109) | ACC :0.589 (0.103) | ACC :0.557 (0.088) | ACC :0.557 (0.09) | ACC :0.579 (0.059) |
|  | SENS :0.293 (0.19) | SENS :0.287 (0.173) | SENS :0.32 (0.148) | SENS :0.293 (0.178) | SENS :0.267 (0.189) | SENS :0.153 (0.141) |
|  | SPEC : 0.711 (0.118) | SPEC : 0.729 (0.144) | SPEC : 0.769 (0.117) | SPEC : 0.733 (0.113) | SPEC : 0.751 (0.148) | SPEC : 0.862 (0.11) |
| **FEATURE SELECTION** | AUC : 0.777 (0.096) | AUC : 0.686 (0.114) | AUC : 0.692 (0.118) | AUC : 0.679 (0.129) | AUC : 0.53 (0.136) | AUC : 0.693 (0.157) |
|  | ACC : 0.723 (0.045) | ACC : 0.669 (0.085) | ACC : 0.677 (0.092) | ACC : 0.672 (0.115) | ACC : 0.595 (0.078) | ACC : 0.704 (0.096) |
|  | SENS : 0.573 (0.134) | SENS : 0.547 (0.192) | SENS : 0.493 (0.191) | SENS : 0.56 (0.205) | SENS : 0.3 (0.156) | SENS : 0.413 (0.236) |
|  | SPEC : 0.822 (0.094) | SPEC : 0.751 (0.119) | SPEC : 0.8 (0.137) | SPEC : 0.747 (0.146) | SPEC : 0.791 (0.145) | SPEC : 0.898 (0.099) |
| **N. OF FEATURES** | 5 | 9 | 2 | 10 | 154 | 2 |
|  |  |  |  |  |  |  |
|  | **BCF** | **BDE** | **BDF** | **CDE** | **CDF** | **ABCD** |
| **ALL FEATURES** | AUC :0.568 (0.117) | AUC :0.479 (0.108) | AUC :0.595 (0.104) | AUC :0.437 (0.132) | AUC :0.575 (0.137) | AUC :0.457 (0.111) |
|  | ACC :0.571 (0.098) | ACC :0.573 (0.078) | ACC :0.581 (0.102) | ACC :0.563 (0.073) | ACC :0.568 (0.102) | ACC :0.547 (0.073) |
|  | SENS :0.32 (0.205) | SENS :0.16 (0.137) | SENS :0.307 (0.168) | SENS :0.127 (0.127) | SENS :0.273 (0.162) | SENS :0.093 (0.125) |
|  | SPEC : 0.738 (0.121) | SPEC : 0.849 (0.133) | SPEC : 0.764 (0.145) | SPEC : 0.853 (0.125) | SPEC : 0.764 (0.119) | SPEC : 0.849 (0.14) |
| **FEATURE SELECTION** | AUC : 0.803 (0.123) | AUC : 0.693 (0.157) | AUC : 0.766 (0.103) | AUC : 0.679 (0.092) | AUC : 0.804 (0.125) | AUC : 0.699 (0.109) |
|  | ACC : 0.733 (0.094) | ACC : 0.704 (0.096) | ACC : 0.699 (0.098) | ACC : 0.669 (0.077) | ACC : 0.736 (0.129) | ACC : 0.677 (0.094) |
|  | SENS : 0.58 (0.217) | SENS : 0.413 (0.236) | SENS : 0.567 (0.226) | SENS : 0.313 (0.207) | SENS : 0.56 (0.244) | SENS : 0.36 (0.198) |
|  | SPEC : 0.836 (0.118) | SPEC : 0.898 (0.099) | SPEC : 0.787 (0.125) | SPEC : 0.907 (0.103) | SPEC : 0.853 (0.125) | SPEC : 0.889 (0.113) |
| **N. OF FEATURES** | 4 | 2 | 4 | 2 | 6 | 2 |
|  |  |  |  |  |  |  |
|  | **ABCE** | **ABCF** | **ABEF** | **ACDE** | **ACDF** | **BCDE** |
| **ALL FEATURES** | AUC :0.467 (0.136) | AUC :0.514 (0.108) | AUC :0.539 (0.132) | AUC :0.418 (0.125) | AUC :0.474 (0.123) | AUC :0.423 (0.109) |
|  | ACC :0.547 (0.082) | ACC :0.531 (0.115) | ACC :0.528 (0.125) | ACC :0.56 (0.073) | ACC :0.523 (0.072) | ACC :0.539 (0.084) |
|  | SENS :0.107 (0.114) | SENS :0.28 (0.187) | SENS :0.273 (0.205) | SENS :0.087 (0.096) | SENS :0.24 (0.164) | SENS :0.107 (0.104) |
|  | SPEC : 0.84 (0.134) | SPEC : 0.698 (0.135) | SPEC : 0.698 (0.128) | SPEC : 0.876 (0.123) | SPEC : 0.711 (0.144) | SPEC : 0.827 (0.144) |
| **FEATURE SELECTION** | AUC : 0.724 (0.137) | AUC : 0.699 (0.139) | AUC : 0.692 (0.139) | AUC : 0.627 (0.118) | AUC : 0.774 (0.123) | AUC : 0.693 (0.157) |
|  | ACC : 0.667 (0.112) | ACC : 0.683 (0.114) | ACC : 0.64 (0.129) | ACC : 0.645 (0.059) | ACC : 0.725 (0.115) | ACC : 0.704 (0.096) |
|  | SENS : 0.38 (0.192) | SENS : 0.493 (0.228) | SENS : 0.433 (0.176) | SENS : 0.227 (0.104) | SENS : 0.493 (0.213) | SENS : 0.413 (0.236) |
|  | SPEC : 0.858 (0.171) | SPEC : 0.809 (0.139) | SPEC : 0.778 (0.163) | SPEC : 0.924 (0.087) | SPEC : 0.88 (0.104) | SPEC : 0.898 (0.099) |
| **N. OF FEATURES** | 2 | 6 | 4 | 16 | 8 | 2 |
|  |  |  |  |  |  |  |
|  | **BCDF** | **BCEF** | **BDEF** | **CDEF** | **ABDF** | **ADEF** |
| **ALL FEATURES** | AUC :0.574 (0.114) | AUC :0.539 (0.142) | AUC :0.581 (0.124) | AUC :0.506 (0.106) | AUC :0.521 (0.138) | AUC :0.494 (0.107) |
|  | ACC :0.576 (0.115) | ACC :0.576 (0.111) | ACC :0.592 (0.115) | ACC :0.557 (0.113) | ACC :0.533 (0.103) | ACC :0.525 (0.121) |
|  | SENS :0.307 (0.204) | SENS :0.313 (0.19) | SENS :0.3 (0.211) | SENS :0.287 (0.192) | SENS :0.28 (0.193) | SENS :0.247 (0.177) |
|  | SPEC : 0.756 (0.13) | SPEC : 0.751 (0.134) | SPEC : 0.787 (0.113) | SPEC : 0.738 (0.15) | SPEC : 0.702 (0.146) | SPEC : 0.711 (0.144) |
| **FEATURE SELECTION** | AUC : 0.829 (0.117) | AUC : 0.659 (0.12) | AUC : 0.653 (0.119) | AUC : 0.659 (0.12) | AUC : 0.791 (0.103) | AUC : 0.653 (0.119) |
|  | ACC : 0.752 (0.129) | ACC : 0.648 (0.119) | ACC : 0.656 (0.116) | ACC : 0.648 (0.119) | ACC : 0.747 (0.098) | ACC : 0.656 (0.116) |
|  | SENS : 0.58 (0.222) | SENS : 0.513 (0.275) | SENS : 0.533 (0.262) | SENS : 0.513 (0.275) | SENS : 0.553 (0.198) | SENS : 0.533 (0.262) |
|  | SPEC : 0.867 (0.118) | SPEC : 0.738 (0.154) | SPEC : 0.738 (0.129) | SPEC : 0.738 (0.154) | SPEC : 0.876 (0.079) | SPEC : 0.738 (0.129) |
| **N. OF FEATURES** | 6 | 1 | 1 | 1 | 4 | 1 |
|  |  |  |  |  |  |  |
|  | **ACFE** | **ABCDE** | **ABCDF** | **ACDEF** | **BCDEF** | **ABDEF** |
| **ALL FEATURES** | AUC :0.477 (0.118) | AUC :0.385 (0.108) | AUC :0.513 (0.129) | AUC :0.45 (0.105) | AUC :0.53 (0.111) | AUC :0.519 (0.13) |
|  | ACC :0.512 (0.099) | ACC :0.472 (0.094) | ACC :0.552 (0.109) | ACC :0.501 (0.112) | ACC :0.555 (0.092) | ACC :0.555 (0.09) |
|  | SENS :0.227 (0.182) | SENS :0.16 (0.129) | SENS :0.293 (0.19) | SENS :0.22 (0.175) | SENS :0.307 (0.181) | SENS :0.28 (0.193) |
|  | SPEC : 0.702 (0.108) | SPEC : 0.68 (0.138) | SPEC : 0.724 (0.148) | SPEC : 0.689 (0.133) | SPEC : 0.72 (0.118) | SPEC : 0.738 (0.113) |
| **FEATURE SELECTION** | AUC : 0.659 (0.12) | AUC : 0.668 (0.142) | AUC : 0.811 (0.129) | AUC : 0.711 (0.1) | AUC : 0.711 (0.1) | AUC : 0.683 (0.121) |
|  | ACC : 0.648 (0.119) | ACC : 0.635 (0.11) | ACC : 0.749 (0.114) | ACC : 0.656 (0.081) | ACC : 0.656 (0.081) | ACC : 0.659 (0.093) |
|  | SENS : 0.513 (0.275) | SENS : 0.433 (0.249) | SENS : 0.593 (0.195) | SENS : 0.547 (0.197) | SENS : 0.547 (0.197) | SENS : 0.52 (0.207) |
|  | SPEC : 0.738 (0.154) | SPEC : 0.769 (0.133) | SPEC : 0.853 (0.125) | SPEC : 0.729 (0.118) | SPEC : 0.729 (0.118) | SPEC : 0.751 (0.145) |
| **N. OF FEATURES** | 1 | 4 | 5 | 7 | 7 | 10 |
|  |  |  |  |  |  |  |
|  | **ABCEF** | **ABCDEF** |  |  |  |  |
| **ALL FEATURES** | AUC :0.5 (0.108) | AUC :0.464 (0.134) |  |  |  |  |
|  | ACC :0.544 (0.103) | ACC :0.496 (0.112) |  |  |  |  |
|  | SENS :0.26 (0.157) | SENS :0.18 (0.188) |  |  |  |  |
|  | SPEC : 0.733 (0.137) | SPEC : 0.707 (0.129) |  |  |  |  |
| **FEATURE SELECTION** | AUC : 0.781 (0.115) | AUC : 0.779 (0.103) |  |  |  |  |
|  | ACC : 0.723 (0.099) | ACC : 0.712 (0.108) |  |  |  |  |
|  | SENS : 0.58 (0.232) | SENS : 0.607 (0.199) |  |  |  |  |
|  | SPEC : 0.818 (0.125) | SPEC : 0.782 (0.128) |  |  |  |  |
| **N. OF FEATURES** | 9 | 4 |  |  |  |  |

Abbreviations: rET = Essential tremor with rest tremor; AUC = Area Under the Curve. The best model is highlighted in bold.
